# The Practical Medicine Series, 1912

**Published:** 1912-12

**Authors:** 


### Books and Magazines.

THE PRACTICAL MEDICINE SERIES, 1912.—We have before us volumes 5, 6 and 7 of this popular annual. Each volume is complete for the year prior to its publication. The set consists of ten volumes, $10; or separate volumes may be ordered at the prices stated.

Volume V.—*Obstetrics.*—Joseph B. DeSeay, A. M., M. D., editor, Northwestern University Medical School, with the collaboration of Herbert M. Stowe, M. D. Price, $1.25.

Volume VI.—*General Medicine.*—Edited by Frank Billings, M. S., M. D., dean Rush Medical College, and J. H. Salisbury, A. M., M. D., Chicago Clinical School. Price, $1.50.

Volume VII.—*Pediatrics; Orthopedic Surgery.*—Pediatrics edited by I. A. Abt, M. D., Northwestern University Medical School, and May Michael, M. D.; orthopedic surgery edited by John Rixlon, A. M., M. D., Rush Medical College, in collaboration with Charles A. Parker, M. D. Price, $1.25. Year Book Publishing Company, Chicago.

This series of publications, which brings the several departments up to date and stands midway between the medical journals and the monographs and standard text-books, has been before the medical profession many years and is well known and a very general favorite. If the reader is not acquainted with it, let him write to the publishers for literature.

